# Left bundle branch pacing: a promising modality for cardiac resynchronisation therapy

**DOI:** 10.1007/s12471-023-01773-4

**Published:** 2023-03-16

**Authors:** Justin G. L. M. Luermans, Kevin Vernooy

**Affiliations:** Department of Cardiology, Cardiovascular Research Institute Maastricht (CARIM), Maastricht University Medical Centre, Maastricht, The Netherlands

Cardiac resynchronisation therapy (CRT) by biventricular pacing (BVP) on top of optimal medical therapy is the mainstay of treatment in patients with heart failure with reduced left ventricular function and left bundle branch block (LBBB) [1]. However, the resynchronisation induced by BVP is non-physiological, as it is the fusion of an epicardial wavefront from the coronary sinus lead and the right ventricular endocardial wavefront. Moreover, BVP might be limited due to unsuccessful coronary sinus lead delivery, suboptimal lead positions, the presence of left ventricular scar tissue, high pacing thresholds, and phrenic nerve stimulation, leading to underuse and non-response [2]. Recently, left bundle branch pacing (LBBP) emerged as a potential alternative to BVP [3]. Several mechanisms explain the capability of LBBP for delivering CRT.

First, Upadhyay et al. showed that the site of conduction block in LBBB is located proximally, at the His or proximal left bundle branch (LBB) level, in 64% of cases [4]. Pacing beyond the site of conduction block in the LBB might correct the LBBB and restore left ventricular synchrony. It has been shown that is possible to engage the conduction system and to capture the LBB by pacing at the left ventricular septal site, since the proximal LBB spreads out beneath the left ventricular subendocardium [3].

Second, pacing at the left ventricular septal site potentially has additional beneficial effects besides the possibility of restoring the LBBB. In healthy human hearts, the ventricular electrical activation starts endocardially on the left interventricular septum [5]. From a mechanistic point of view, it makes sense to restore the physiological electrical activation sequence by pacing at the earliest activated left ventricular site in patients with heart failure induced by LBBB. Pacing the left ventricular septal endocardial site captures fast conducting superficial fibres [6] and bypasses slow leftward transseptal conduction, which is responsible for a considerable part of the total dyssynchrony in LBBB [7]. Circumferential left ventricular endocardial conduction, left ventricular activation from endocardium to epicardium, and simultaneous right ventricular activation theoretically reduces total activation time. With these mechanistic hypotheses in mind, left ventricular septal pacing and BVP were compared in 27 patients with a CRT indication [8]. Left ventricular septal pacing resulted in short-term haemodynamic improvement and electrical resynchronisation that was at least as good as in BVP [8].

In this issue, Rademakers et al. describe the safety, feasibility and outcomes of LBBP in 40 CRT patients compared with an equally sized historical control group treated with BVP. LBBP was successful in 31 patients and no LBBP-related complications occurred. LBBP resulted in a greater reduction in QRS duration compared with BVP and a comparable clinical and echocardiographic improvement. Although these results seem promising, we need to emphasise the current limitations of LBBP as an alternative to CRT.

First, the success rate of achieving LBBP, as described by Rademakers et al., is still limited (78%), which is comparable with earlier reports in which LBBP consistently was performed by experienced operators [9]. Limited success rates, especially in heart failure patients, might be associated with the current lack of dedicated implantation tools and materials for performing LBBP, as well as with patient factors. The inability to penetrate the septum, for instance due to scar tissue, is a frequently encountered problem.

Second, most studies to date were performed in patients with nonischaemic cardiomyopathy and typical LBBB. Rademakers et al. also predominantly included patients with nonischaemic cardiomyopathy with LBBB according to Strauss criteria. This group historically shows the best response to CRT. Consequently, LBBP might be less beneficial in patients with ischaemic cardiomyopathy, in whom distal conduction delay is often present. Corrective pacing beyond the site of block seems impossible in the case of atypical LBBB and distal conduction system disease [4]. Selection of patients with proximal LBBB seems, therefore, important, but the site of block cannot always be reliably predicted noninvasively.

Third, engaging the conduction system may be difficult when pacing the left ventricular septum, and defining the presence of LBB capture can be challenging, especially in patients with distal conduction abnormalities. LBB capture criteria are not solid and still in development [10]. Moreover, currently even the additional benefit of obtaining LBB capture on top of pacing the left ventricular septum is unclear.

We should be aware that heart failure patients are a heterogeneous group of patients, some of them having focal LBBB which is correctable with LBBP, and some of them having more complex and distal conduction system disease which could, in part, be addressed by performing LBBP in combination with left ventricular coronary sinus pacing. The future of CRT lies in understanding the underlying dyssynchrony mechanisms and selecting the appropriate combination of CRT modalities.

As there have been no large clinical trials comparing LBBP with BVP in CRT patients, LBBP is not recommended as a first-line treatment yet in the current ESC guidelines [1]. Therefore, applying LBBP in clinical practice at this time as a first-line treatment should be prudently considered, although it seems a promising strategy for the future.

## References

[1] Glikson M, Nielsen JC, Kronborg MB (2021). 2021 ESC guidelines on cardiac pacing and cardiac resynchronization therapy. Eur Heart J.

[2] Daubert C, Behar N, Martins RP (2017). Avoiding non-responders to cardiac resynchronization therapy: a&nbsp;practical guide. Eur Heart J.

[3] Huang W, Su L, Wu S (2017). A novel pacing strategy with low and stable output: pacing the left bundle branch immediately beyond the conduction block. Can J Cardiol.

[4] Upadhyay GA, Cherian T, Shatz DY (2019). Intracardiac delineation of septal conduction in left bundle-branch block patterns. Circulation.

[5] Durrer D, van Dam RT, Freud GE (1970). Total excitation of the isolated human heart. Circulation.

[6] Myerburg RJ, Gelband H, Nilsson K (1978). The role of canine superficial ventricular muscle fibers in endocardial impulse distribution. Circ Res.

[7] Strik M, van Deursen CJ, van Middendorp LB (2013). Transseptal conduction as an important determinant for cardiac resynchronization therapy, as revealed by extensive electrical mapping in the dyssynchronous canine heart. Circ Arrhythm Electrophysiol.

[8] Salden F, Luermans J, Westra SW (2020). Short-term hemodynamic and electrophysiological effects of cardiac resynchronization by left ventricular septal pacing. J Am Coll Cardiol.

[9] Jastrzebski M, Kielbasa G, Cano O (2022). Left bundle branch area pacing outcomes: the multicentre European MELOS study. Eur Heart J.

[10] Huang W, Chen X, Su L (2019). A beginner’s guide to permanent left bundle branch pacing. Heart Rhythm.

